# Feasibility Study on Predicting Recurrence Risk of Bladder Cancer Based on Radiomics Features of Multiphase CT Images

**DOI:** 10.3389/fonc.2022.899897

**Published:** 2022-06-02

**Authors:** Jing Qian, Ling Yang, Su Hu, Siqian Gu, Juan Ye, Zhenkai Li, Hongdi Du, Hailin Shen

**Affiliations:** ^1^ Department of Radiology, The First Affiliated Hospital of Soochow University, Suzhou, China; ^2^ Department of Radiology, Suzhou Kowloon Hospital Shanghai Jiao Tong University School of Medicine, Suzhou, China

**Keywords:** bladder cancer, recurrence, multiphase CT images, radiomics, retrospective studies

## Abstract

**Background:**

Predicting the recurrence risk of bladder cancer is crucial for the individualized clinical treatment of patients with bladder cancer.

**Objective:**

To explore the radiomics based on multiphase CT images combined with clinical risk factors, and to further construct a radiomics-clinical model to predict the recurrence risk of bladder cancer within 2 years after surgery.

**Methods:**

Patients with bladder cancer who underwent surgical treatment at the First Affiliated Hospital of Soochow University from January 2016 to December 2019 were retrospectively included and followed up to record the disease recurrence. A total of 183 patients were included in the study, and they were randomly divided into training group and validation group in a ratio of 7: 3. The three basic models which are plain scan, corticomedullary phase, and nephrographic phase as well as two combination models, namely, corticomedullary phase + nephrographic phase and plain scan + corticomedullary phase + nephrographic phase, were built with the logistic regression algorithm, and we selected the model with higher performance and calculated the Rad-score (radiomics score) of each patient. The clinical risk factors and Rad-score were screened by Cox univariate and multivariate proportional hazard models in turn to obtain the independent risk factors, then the radiomics-clinical model was constructed, and their performance was evaluated.

**Results:**

Of the 183 patients included, 128 patients constituted the training group and 55 patients constituted the validation group. In terms of the radiomics-clinical model constructed by three independent risk factors—number of tumors, tumor grade, and Rad-score—the AUCs of the training group and validation group were 0.813 (95% CI 0.740–0.886) and 0.838 (95% CI 0.733–0.943), respectively. In the validation group, the diagnostic accuracy, sensitivity, and specificity were 0.727, 0.739, and 0.719, respectively.

**Conclusion:**

Combining with radiomics based on multiphase CT images and clinical risk factors, the radiomics-clinical model constructed to predict the recurrence risk of bladder cancer within 2 years after surgery had a good performance.

## 1 Introduction

Bladder cancer is one of the most common malignant tumors of the urinary system (1, 2). High recurrence rate is a serious problem faced by the treatment of bladder cancer (3, 4). Bladder cancer can be divided into non-muscle-invasive bladder cancer (NMIBC) and muscle-invasive bladder cancer (MIBC) (5). According to the 2021 EAU scoring model (1), patients with non-muscle-invasive bladder cancer are stratified as having low, intermediate, high, or very high risk on the basis of their individual risk of progression. Moreover, patients with high-risk or very high-risk NMIBC still have a high recurrence rate and easily progress to MIBC after transurethral resection of bladder tumor (TURBT) (6). Local or metastatic recurrence occurs within the next 2 years in approximately 5%–50% of patients with muscle-invasive bladder cancer, even though the radical cystectomy (RC) can remove most of the diseased tissues (5). For patients with bladder cancer at high recurrence risk, a more effective treatment at an early stage and a closer follow-up management strategy are expected to increase their survival. Therefore, the accurate prediction of recurrence risk of bladder cancer is crucial for the prediction and individualized treatment of patients with bladder cancer.

At present, the risk tables developed by the European Organization for Research and Treatment of Cancer (EORTC) and the Club Urológico Español de Tratamiento Oncológico (CUETO) are widely used in clinical practice (7), but the accuracy of both is less than 0.7 (7–10). Therefore, it is urgent to establish a new model to predict the recurrence risk of bladder cancer.

In recent years, radiomics has shown great potential for disease diagnosis and prediction with the continuous development of imaging technology (11–13). Radiomics is an emerging technology that converts medical images into high-dimensional mineable data through feature engineering and machine learning techniques. These information can supplement other relevant data, such as clinical parameters and pathological factors, so as to complete the construction of the non-invasive quantitative diagnosis of tumor, therapeutic effect evaluation, and prognostic prediction model (14, 15). According to the early literatures, the radiomics features of CT or MRI are of great value in tumor grading, evaluation of lymph node metastasis, muscle invasion, differentiation, and recurrence prediction (16, 17). For example, the study of Del Giudice Francesco et al. (18) indicates that the diagnostic of VI-RADS score 5 in predicting locally advanced bladder cancer is of great accuracy, and it can help to identify those patients who could avoid the morbidity of deep TURBT in favor of histologic sampling-TUR before radical cystectomy (RC). It is also found that multiparametric MRI has good performance in predicting the recurrence risk of patients with bladder cancer (19, 20). In the study conducted by Xu et al. (20), a radiomics-clinical model was built by using the patient’s Rad-score and tumor staging as the independent risk factors based on the radiomics features extracted by preoperative multiparametric MRI combined with important clinical risk factors, which had a good performance for the postoperative and preoperative recurrence risk-stratified prediction of patients within 2 years. Specifically, the AUC was 0.915 for the efficiency training set and 0.838 for the validation set. However, it is known that the feasibility of multiphase CT images in predicting the recurrence risk of patients with bladder cancer has not been fully investigated.

Based on these findings, we can hypothesize that 1) small changes in tissues can be discriminated based on the radiomics features extracted from multiphase CT images, which may predict the recurrence of bladder cancer, and that 2) radiomics combined with clinical pathological information, such as age, gender, tumor grade, tumor staging, number of tumors, and surgical method, may improve the efficacy of bladder cancer recurrence prediction.

## 2 Materials and Methods

### 2.1 Patients

**Figure 1:**
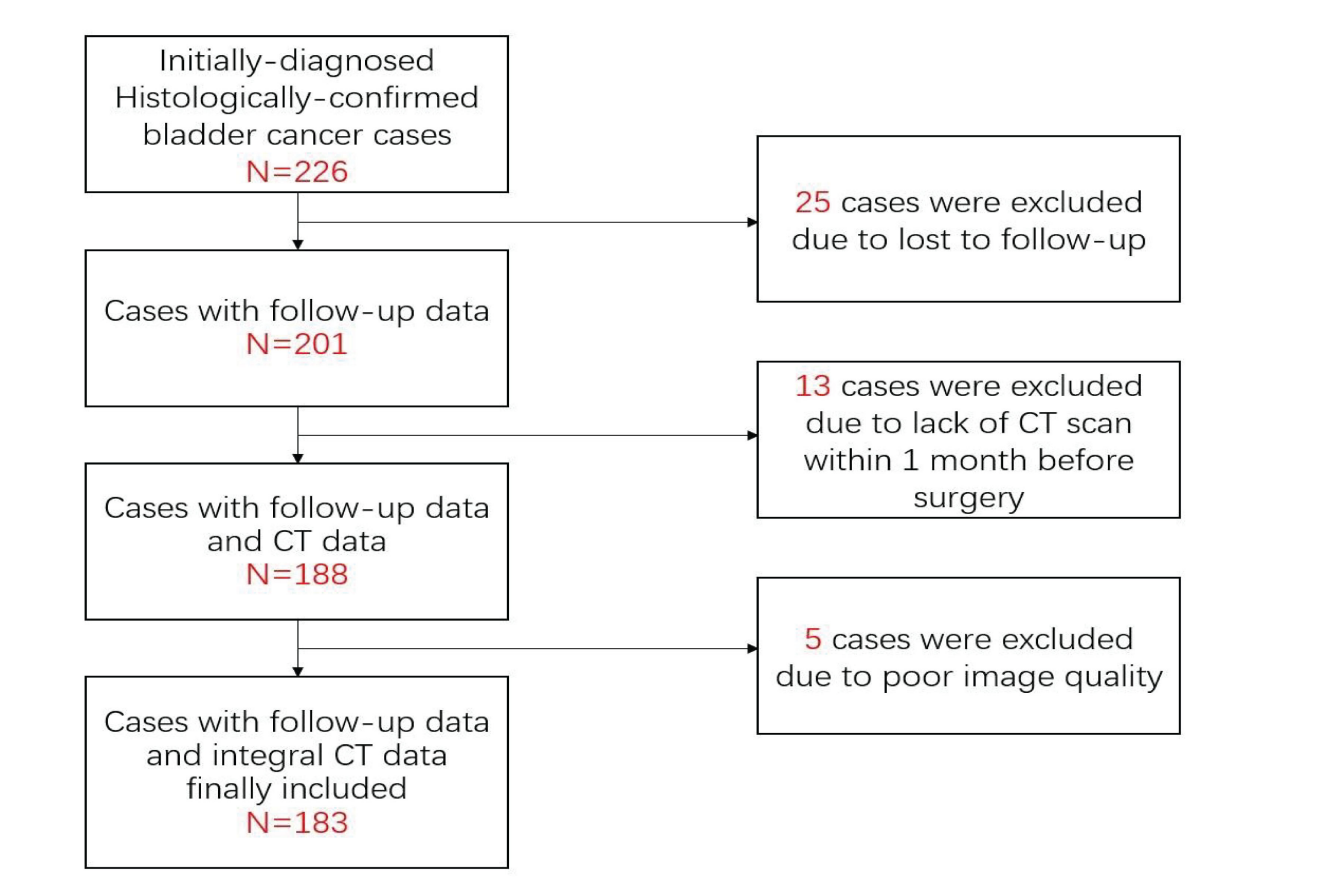
Flowchart shows selection criteria for the 183 patients in the study group.

This retrospective study was approved by the Institutional Review Committee. A retrospective search was performed in the pathology database and radiology database to identify patients with bladder cancer at the First Affiliated Hospital of Soochow University between January 2016 and December 2019. The criteria for inclusion are as follows: (1) complete pathological data are provided; (2) the images of CT urography (CTU) used to evaluate the lesion are complete; (3) CT scan is performed within 30 days before surgery. The exclusion criteria are as follows: (1) CT images are unqualified; (2) lesions of which the boundaries are difficult to define, such as only the lesion considered as abnormal enhancement, without intracavitary lump or bladder wall thickening; (3) clinicopathological data are missing or incomplete (**Figure 1**).

### 2.2 Research Method

#### 2.2.1 Clinical Data Collection

The clinical information of the patients of the First Affiliated Hospital of Soochow University from January 2016 to December 2019, such as age, gender, histopathological grade, tumor size in bladder cavity, number of tumors, and surgical method, have been collected.

#### 2.2.2 Patient Follow-up and Results

All patients were followed up once within the 3–5 months after surgery, then every 6 months for the next 2 years, and annually thereafter. Follow-up content includes the cystoscopy and radiological examination. Recurrence occurs when a bladder tumor reappears in the bladder, prostate glands, urethra, pelvis, or ileum after surgery. The patient’s relapse-free survival (RFS) is recorded from the previous surgery until the date of the confirmed tumor recurrence. During the 2-year follow-up, bladder cancer recurrence confirmed by cystoscope and pathology examination was defined as recurrence group, and the rest were no recurrence groups.

#### 2.2.3 CT Image Acquisition and Region of Interested Segmentation

A Siemens CT scanner (Somatom Definition Flash), Toshiba CT scanner (Aquilion ONE), and Philips CT scanner (Philips Ingenuity) were used. The scanning parameters are as follows: tube voltage 100–120 kVp, automatic tube current in mA/s, FOV 500 mm, and a scanning layer thickness of 5–6 mm. For enhanced scanning, the contrast agent iohexol (350 mg I/ml) is injected *via* the elbow vein at an injection rate of 3 ml/s. After the locating image was collected at the supine position, plain scanning and three-phase dynamic enhanced scanning were performed for the patient. The dynamic enhanced scanning of the corticomedullary phase, nephrographic phase, and excretory phase was performed after 30, 60, and 150 s, respectively, after the contrast agent was injected. All images were treated with the standard reconstitution method. The interlamellar spacing and layer thickness were both 5–6 mm. In this study, the plain scan, corticomedullary phase, and nephrographic phase will be used for subsequent studies.

**Figure 2:**
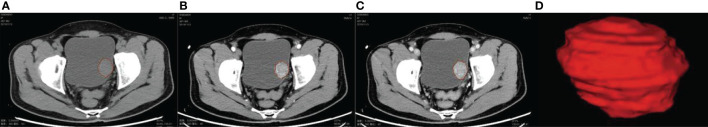
**(A–C)** Red curves show the tumor contour of the plain scan, corticomedullary phase, and nephrographic phase, respectively; **(D)** is the generated tumor 3D-VOI.

Two radiologists A and B (with experience in imaging diagnosis of urinary system diseases for 7 and 13 years, respectively) completed the image reading of plain scan, corticomedullary phase, and nephrographic phase together without knowing the patient’s condition. In case of disagreement, it was resolved through negotiation. The 3D tumor lesion ROI was semiautomatically delineated with a Deepwise scientific research platform (http://label.deepwise.com). In the process of ROI delineation in the focal area, automatic delineation was performed based on the partitioning algorithm of the level set at first, and then the radiologist manually corrected the wrong partitioning. If multiple lesions were encountered, only the largest lesions were delineated because the pathology results show the largest tumor within the bladder (**Figure 2**). Thirty lesions were randomly selected and re-segmented by a senior radiologist (reader B) to calculate the inter-class correlation coefficient (ICC).

#### 2.2.4 Feature Extraction and Selection

Pyradiomics 3.0.1 was used to extract the radiomics features within the ROI of tumor lesions. After each tumor VOI underwent wavelet filtering, Laplace transformation, and calculation of image intensity square, the original image or preprocessed image was extracted. Based on the original images and the filtered images of plain scan, corticomedullary phase, and nephrographic phase, 910 radiomics features were extracted, including 180 first-order features describing the statistical distribution of CT values within the tumor, and 220 features describing the gray-level cooccurrence matrix (GLCM) features of tumor texture, 160 gray-level size zone matrix (GLSZM), 160 gray-level run length matrix (GLRLM) features, 140 gray-level dependence matrix (GLDM) features, and 50 features of neighboring gray tone difference matrix (NGTDM) features. All extracted radiomics features are subjected to Z-score normalization, and the formula for feature normalization is z = (x-mean)/std. Among all the features in each model of the plain scan, corticomedullary phase, and nephrographic phase, the lowest P value was filtered out with the F test for follow-up analysis after calculating the linearly dependent coefficient.

#### 2.2.5 Construction of the Radiomics Prediction Model

The radiomics feature subset was optimized based on 10 random splits of 7:3; the three basic models plain scan, corticomedullary phase, and nephrographic phase as well as two combination models were established with the logistic regression algorithm. The application performance indicators include AUC, accuracy, sensitivity, and specificity to evaluate the predictive performance of its radiomics model. The specific method was used as follows: divide the data into 10 parts randomly, with 7 of them used as the training set in turn, and the remaining 3 as the validation set. The corresponding model prediction probability can be obtained for each test, and the mean of the 10 prediction results is used for the estimate of accuracy; a total of 10 times of 7:3 random split verifications were performed, and the mean value of each efficiency index prediction was calculated as the final evaluation result of model efficiency. The AUC values of the five models were compared with Delong’s test, and the radiomics model with higher performance was selected for subsequent analysis.

#### 2.2.6 Radiomics-Clinical Model Construction and Its Efficacy Evaluation

First, the optimal radiomics features screened by the radiomics model with higher performance were put into a logistic model to obtain the regression coefficients, and then the Rad-score of each patient can be calculated with the linear combination of these features and their corresponding regression coefficients. Finally, the clinical risk factors and Rad-score were screened through Cox univariate analysis, the variables with significant statistical differences were retained (P < 0.05), and Cox multivariate analysis was further performed to determine the independent risk factors (P < 0.05). Then, the radiomics-clinical model for predicting recurrence of bladder cancer was built with these independent risk factors. The performance was evaluated with the calibration curve and AUC of the training group and the validation group. Finally, the clinical benefit of the radiomics-clinical model was further validated using decision curve analysis.

### 2.3 Statistical Analysis

Statistical analysis was performed using SPSS 25.0 and R 4.1.0 software. The counting data were compared with the chi-square test. For measurement data, the Kolmogorov–Smirnov test was performed to test whether it is normal distribution at first. If the normal distribution is conformed, an independent sample t-test was conducted for comparison, which was expressed as the mean ± standard deviation. If the normal distribution is not conformed, the Mann–Whitney U test will be used and expressed as mean (P25, P75). When P < 0.05, the difference is statistically significant.

## 3 Results

### 3.1 General Clinical Data

A total of 183 patients with bladder cancer who met the inclusion and exclusion criteria were included in this study, including 160 men and 23 women; the mean age was (67.41 ± 11.44) years.

During the follow-up on December 31, 2021, there were 76 patients with recurrence (Recurrence group) and 107 patients without recurrence (No recurrence group). The median RFS for all recurrent patients was 10.5 months, with a range of 1–24 months, while the median RFS for all non-recurrent patients was 33 months, with a range of 24–67 months. The clinical data of patients of the recurrence group and no recurrence group are shown in **Table 1**.

**Table 1:** Comparison of clinical data between the recurrence group and no recurrence group.

Characteristics	Recurrence	No recurrence	χ2/t	P
n	76	107		
Gender, n (%)			0.041	0.839
Male	66 (36.1%)	94 (51.4%)		
Female	10 (5.5%)	13 (7.1%)		
Age, n (%)			3.745	0.053
>65	52 (28.4%)	58 (31.7%)		
≤65	24 (13.1%)	49 (26.8%)		
Grade, n (%)			4.822	0.028
Low	28 (15.3%)	57 (31.2%)		
High	48 (26.2%)	50 (27.3%)		
Staging, n (%)			5.002	0.025
<T2	61 (33.3%)	98 (53.6%)		
≥T2	15 (8.2%)	9 (4.9%)		
Number of tumors, n (%)			6.184	0.013
Single	38 (20.8%)	73 (39.9%)		
Multiple	38 (20.8%)	34 (18.6%)		
Surgical method, n (%)			0.036	0.850
Nonradical cystectomy	69 (37.7%)	98 (53.6%)		
Radical cystectomy	7 (3.8%)	9 (4.9%)		
Tumor size (mm), median	23.5 (16.75, 37.25)	17 (13, 24)	3.874	< 0.001
RFS (month), median	10.5 (6, 15)	33 (26, 39)		< 0.001

### 3.2 Consistency Test for Feature Extraction

The average ICC was 0.897 for the plain scan model features, 0.928 for the corticomedullary phase model features, and 0.860 for the nephrographic phase model features, which showed good consistency among the observers.

### 3.3 Radiomics Feature Screening and Establishment of the Prediction Model

Based on the original images and the filtered images, 614 features were removed and a total of 296 features were retained in the 910 features of each model of plain scan, corticomedullary phase, and nephrographic phase after the linearly dependent coefficient was calculated. The 16 features with the lowest P-values were filtered out using an F-test for the follow-up analysis.

### 3.4 Performance Evaluation of the Radiomics Prediction Model

**Figure 3:**
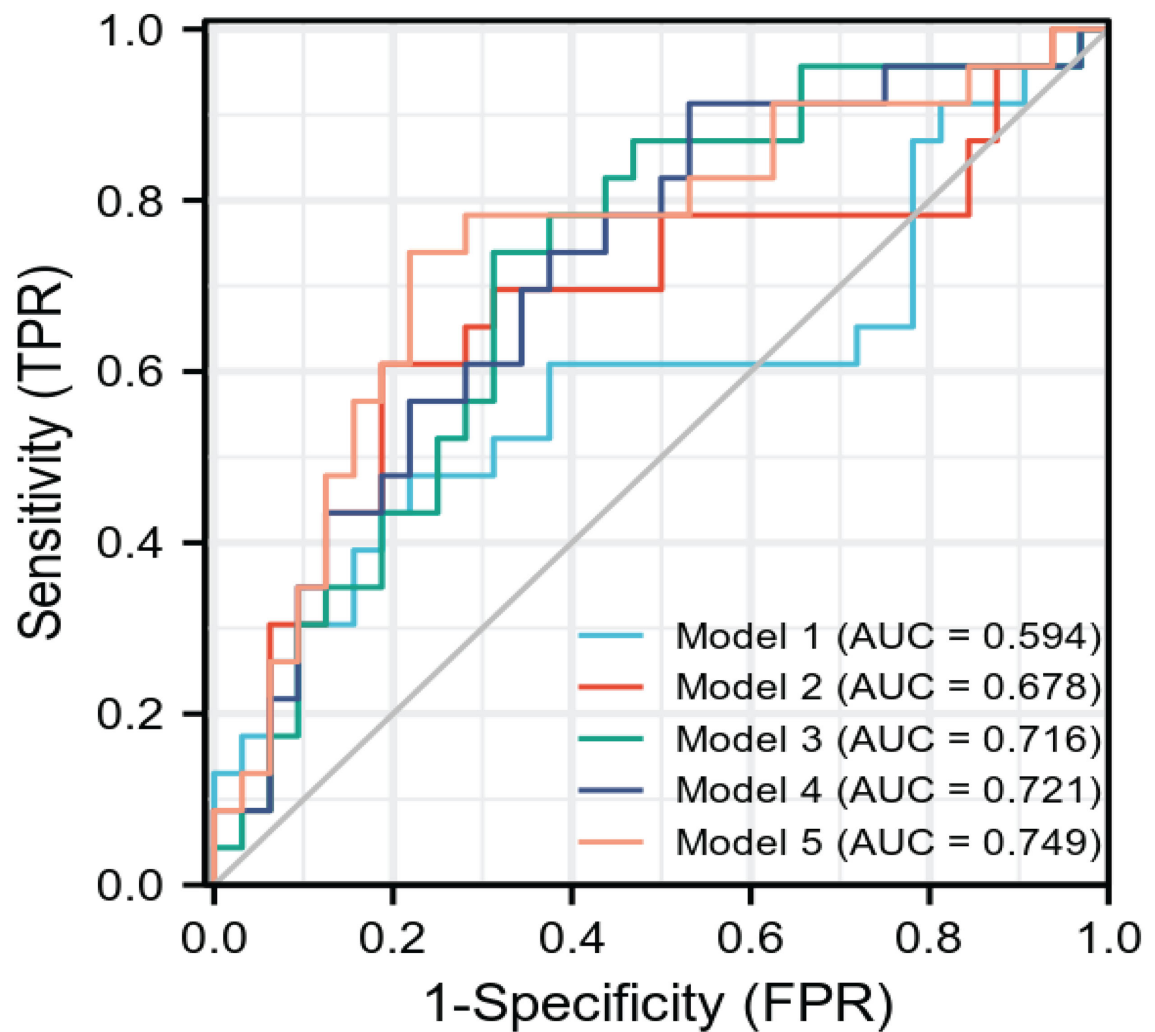
The ROC curve of each model radiomics model. AUC is the same as **Table 2**. ROC, receiver operating characteristics.

After the radiomics feature screening was completed, the radiomics feature subset was optimized based on 10 times of 7:3 random splits, and the three basic models plain scan, corticomedullary phase, and nephrographic phase as well as two combination models, namely, corticomedullary phase + nephrographic phase and plain scan + corticomedullary phase + nephrographic phase, were established with the logistic regression algorithm. The AUC of the plain scan, corticomedullary phase, nephrographic phase, and the corticomedullary phase + nephrographic phase and plain scan + corticomedullary stage + nephrographic stage models for predicting recurrence of bladder cancer within 2 years after surgery were 0.594, 0.678, 0.716, 0.721, and 0.749, respectively (**Table 2** and **Figure 3**). Among the five radiomics models, the combination model of plain scan + corticomedullary phase + nephrographic phase showed the best predictive performance, and so it was used for the subsequent construction of the radiomics-clinical model (**Table 3**). Delong’s test shows that there is a statistical difference (P = 0.044) in the validation set AUC between the plain scan model and the combination model of plain scan + corticomedullary phase + nephrographic phase, but no statistical difference between the other models (all P > 0.05) (**Table 3**).

**Table 2:** The diagnostic performance of radiomics model in the training group and validation group.

Radiomics model	Training group				Validation group			
	AUC (95% CI)	Accuracy	Sensitivity	Specificity	AUC (95% CI)	Accuracy	Sensitivity	Specificity
Model 1	0.726 (0.636–0.815)	0.672	0.604	0.720	0.594 (0.430–0.758)	0.618	0.609	0.625
Model 2	0.794 (0.716–0.872)	0.711	0.679	0.733	0.678 (0.523–0.833)	0.727	0.609	0.813
Model 3	0.778 (0.698–0.857)	0.703	0.755	0.667	0.716 (0.577–0.855)	0.673	0.826	0.563
Model 4	0.852 (0.788–0.917)	0.734	0.717	0.747	0.722 (0.583–0.860)	0.636	0.609	0.656
Model 5	0.811 (0.736–0.886)	0.719	0.698	0.733	0.749 (0.611–0.887)	0.696	0.696	0.781

**Table 3:** P values of the five radiomics model obtained by Delong’s test.

Variable 1	Variable 2	P
Model 1	Model 2	0.337
Model 1	Model 3	0.185
Model 1	Model 4	0.115
Model 1	Model 5	0.044
Model 2	Model 3	0.677
Model 2	Model 4	0.542
Model 2	Model 5	0.208
Model 3	Model 4	0.946
Model 3	Model 5	0.688
Model 4	Model 5	0.529

(Model 1: plain scan, Model 2: corticomedullary phase, Model 3: nephrographic phase, Model 4: corticomedullary phase + nephrographic phase, Model 5: plain scan+ corticomedullary phase + nephrographic phase).

**Figure 4:**
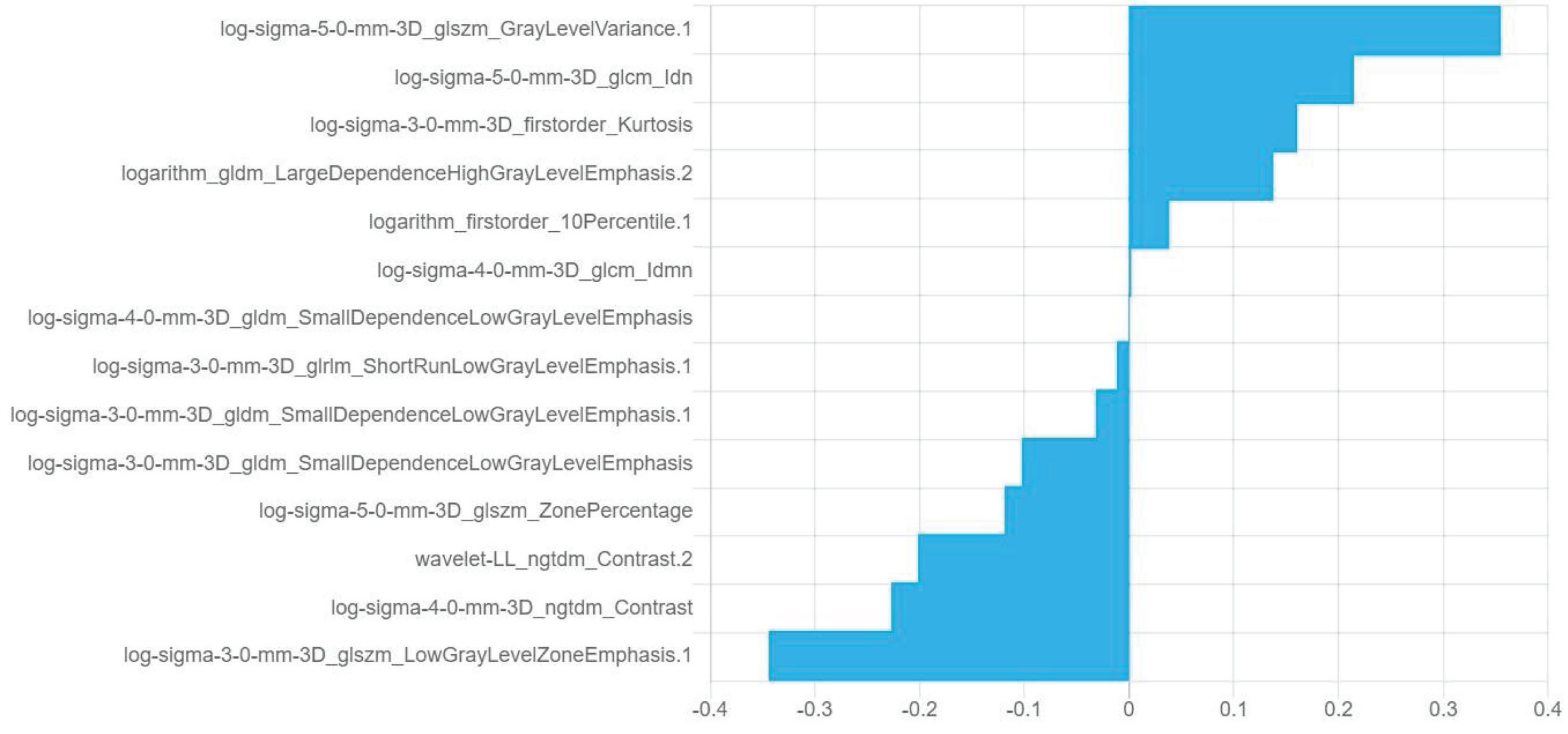
Information and corresponding feature weights of the 14 optimal features screened by the plain scan + corticomedullary phase + nephrographic phase model.

Fourteen optimal radiomics features were retained in the combination model of plain scan + corticomedullary phase + nephrographic phase constructed using the logistic regression algorithm (**Figure 4**). Among the 14 optimal omics features, there are two first-order features, two gray-level cooccurrence matrix features, four gray-level dependence features, three gray-level size zone matrix features, one gray-level run-length matrix feature, and two neighborhood gray-level difference matrix features.

### 3.5 Radiomics-Clinical Model Construction and Efficiency Evaluation

**Figure 5:**
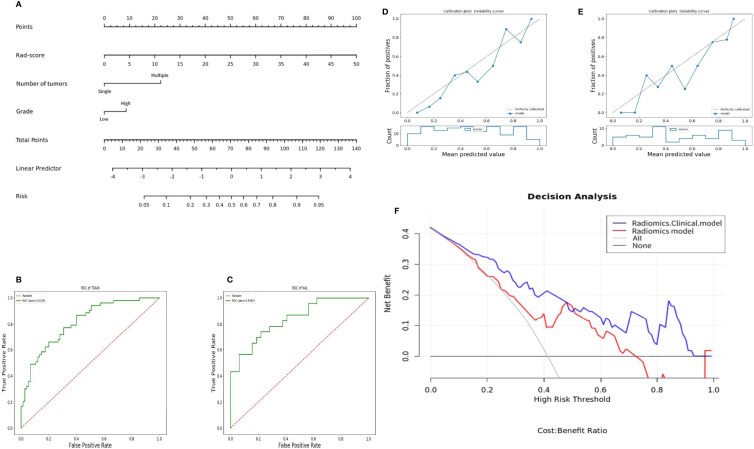
Nomogram and its diagnostic performance: **(A)** Nomogram constructed based on patient number of tumors, tumor grade, and Rad-score; **(B)** and **(C)** are the ROC curves of the training group and the validation group of the radiomics-clinical model; **(D)** and **(E)** are the calibration curves of the nomogram of the training group and the validation group; **(F)** decision curve of the radiomics-clinical and radiomics model predicts the net income increment in recurrence risk of bladder cancer within 2 years after surgery.

**Figure 6:**
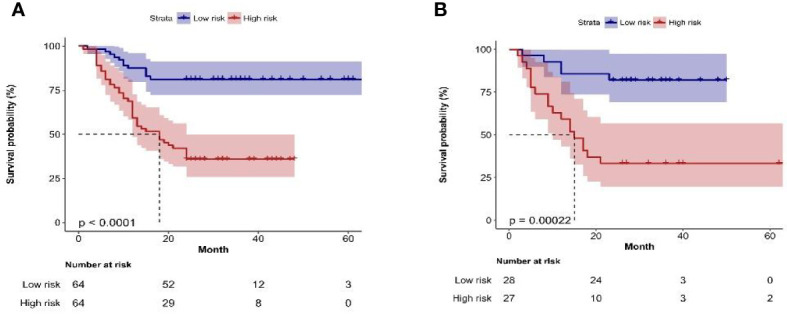
**(A, B)** Kaplan–Meier plots of bladder cancer within 2 years after surgery in the training group and the validation group constructed by recurrence risk stratification based on nomogram.

After selecting 14 optimal radiomics features, the logistic model was used to linearly combine these features into one feature, a rad score formula was established, and the Rad-score of each patient was calculated. Rad-score and clinicopathologic risk factors were analyzed by using Cox univariate and multivariate proportional hazard models, as shown in **Table 4**. Among them, tumor grade, tumor staging, number of tumors, tumor size, and Rad-score significantly correlated with the recurrence of bladder cancer (P < 0.05). In multivariate analysis, the number of tumors (HR 2.428, 95% CI 1.513–3.895, P < 0.001), tumor grade (HR 1.843, 95% CI 1.094–3.104, P = 0.022), and Rad-Score (HR 1.107, 95% CI 1.073–1.143, P < 0.001) were independent risk factors for recurrence of bladder cancer. **Figure 5A** shows the radiomics-clinical model and nomogram constructed with three independent risk factors. The AUC is 0.813, the accuracy is 0.711, the sensitivity is 0.700, and the specificity is 0.720 for the training group, while the AUC, accuracy, sensitivity, and specificity were 0.838, 0.727, 0.739, and 0.719, respectively, for the validation group (**Figures 5B, C**). According to the nomogram, if a patient has multiple tumors of high grade, and the Rad-score is 25, the three-point scores will be close to 21, 8, and 50, respectively, and the “total score” is 79 points, which is equivalent to the “recurrence risk” about 0.68 points. The calibration curves with the training and validation cohorts are depicted in **Figures 5D, E**. The calibration curves show a good agreement between the predicted probabilities of the radiomics-clinical model and the actual bladder cancer recurrence within 2 years after surgery, and the decision curve analysis of different models is shown in **Figure 5F**. Decision curve analysis shows that the clinical application value of the radiomics-clinical model is higher than that of the radiomics model. The median recurrence risk (0.447) obtained from patients in the training cohort was used, and then all patients were divided into high recurrence risk group and low recurrence risk group. **Figure 6** shows a Kaplan–Meier (KM) plots in two cohorts. As shown in **Table 5**, the comprehensive prediction performance of the radiomics-clinical model is better than that of the radiomics model, with the C-index of 0.751 (95% CI 0.718–0.784) and 0.750 (95% CI 0.704–0.795), respectively, in the training group and validation group.

**Table 4:** Cox univariate and multivariate proportional hazard models of risk factors for recurrence of bladder cancer.

Factors	Univariate		Multivariate	
	HR (95% CI)	P	HR (95% CI)	P
Gender	1.058 (0.544–2.058)	0.867		
Age	0.634 (0.391–1.029)	0.065		
Grade	1.732 (1.087–2.762)	**0.021**	1.843 (1.094–3.104)	**0.022**
Staging	1.882 (1.069–3.314)	**0.028**	0.822 (0.429–1.573)	0.554
Number of tumors	1.739 (1.109–2.727)	**0.016**	2.428 (1.513–3.895)	**<0.001**
Surgical method	0.993 (0.456–2.161)	0.986		
Tumor size	1.029 (1.015–1.043)	**<0.001**	0.987 (0.966–1.008)	0.232
Rad-score	1.086 (1.061–1.112)	**<0.001**	1.107 (1.073–1.143)	**<0.001**

Bold indicates statistical significance at the level of P <0.05.

**Table 5:** Performance of evaluating patients’ RFS in different models of training groups and validation groups.

Model	C-index (95% CI)
	Training group
Radiomics model	0.748 (0.714–0.781)
Radiomics-clinical model	0.751 (0.718–0.784)
	Validation group
Radiomics model	0.670 (0.613–0.727)
Radiomics-clinical model	0.750 (0.704–0.795)

## 4 Discussion

High recurrence rate is a significant epidemiological feature of bladder cancer (21). Cystoscopy is an important reference for the preoperative diagnosis of bladder cancer (22). However, it is an invasive examination with a certain bladder perforation risk. More importantly, due to the heterogeneity of lesions, there may be a misdiagnosis for cystoscopy resulting in the wrong estimation of recurrence risk based on the EORTC prediction model, thereby delaying the subsequent treatment of bladder cancer.

With the continuous development of image technology, imaging diagnosis has played an increasingly important role in the early diagnosis of diseases, curative effect monitoring, and prognosis evaluation. In recent years, MRI has been recognized as an important tool for assessing bladder cancer (23), because MRI has a high resolution of soft tissues. The advent of the novel VI-RADS has certainly renovated the clinical impact of clinical imaging for bladder cancer staging purposes (24). What is more, the multiparametric MRI of the bladder with VI-RADS scores might become a tool for clinical doctors to tailor therapeutic planning according to patients’ risk as per precision personalized medicine. VI-RADS has excellent inter-reader agreement to discriminate NMIBC from MIBC, which stresses the importance that standardization and reproducibility of VI-RADS may confer to multiparametric magnetic resonance for preoperative bladder cancer staging. Since VI-RADS is a promising diagnostic tool, the accuracy of VI-RADS for evaluating bladder cancer staging varies because it is highly dependent on the experience of radiologists, which requires a subjective reviewing process. It is difficult for radiologists to distinguish muscle layers from non-muscle layers on CT images with naked eyes. However, the appearance of radiomics has made it possible by high-throughput extracting image features related to disease. Moreover, the technology of radiomics can guarantee a good inter-observer agreement by feature normalization and feature selection with high ICCs. In our study, the mean ICCs of these features extracted from three models were both over 0.85. In other radiomics studies, Zhang et al. (25) filtered out the features with ICCs less than 0.8 in order to enhance the reproduction of the model and conducted a CT-based radiomics model, which has a good performance in predicting muscle invasiveness of bladder cancer before surgery. The AUCs in the internal and external testing cohorts are 0.820 (95% CI 0.698–0.941) and 0.784 (95% CI 0.674–0.893), respectively, considering the high price of MRI examination, slow scanning, low popularity of equipment, long appointment time, and contraindications of scanning. Some patients with bladder cancer are not suitable for MRI examination. On the contrary, CT examination has the advantages of low price, fast scanning, and wide popularity of equipment. Therefore, CT-based radiomics of bladder cancer-related research results have a good prospect.

Up to now, the application value of CT radiomics technology in the diagnosis and treatment of bladder cancer in relevant studies has been reported. Regarding the prediction of bladder cancer grade, Zhang et al. (26) included 145 patients with bladder cancer and divided them into a training set (108 cases) and a validation set (37 cases). Based on enhanced CT images, a radiomics model was constructed which identified bladder cancer with high and low pathology with AUC being 0.860 and the sensitivity and specificity being 0.885 and 0.727, respectively. Regarding the evaluation on efficacy of bladder cancer after neoadjuvant therapy, Cha et al. (27) showed that for patients with muscle-invasive bladder cancer, preoperative neoadjuvant therapy and computer decision-making system based on CT radiomics can improve the recognition ability of patients with complete response to neoadjuvant chemotherapy.

Relatively more studies were performed on CT radiomics in the aspects of bladder cancer grade, staging, invasion, and efficacy, but few related studies on predicting recurrence risk of bladder cancer based on CT radiomics were reported. For example, in the preliminary study conducted by Zhang et al. (28) based on CT radiomics, the AUCs of several models for predicting the recurrence of bladder cancer within 1 year after surgery were all between 0.70 and 0.73. However, there were no clinical risk factors incorporated in this study and therefore the effect on clinical decision-making was limited.

Nevertheless, the abovementioned literatures are all based on nephrographic phase CT images for bladder cancer research, and few studies are conducted based on multiphase CT images; whether the performance of prediction model can be improved is still unknown.

Therefore, in this study, the logistic regression algorithm was used to construct three basic models plain scan, corticomedullary phase, and nephrographic phase of the single-phase CT image, as well as two combination models of multiphase CT images, and evaluate the performance of the training group and validation group with the best performance of the combination model of plain scan + corticomedullary phase + nephrographic phase. The AUC is 0.811 for the training group and 0.749 for the validation group, indicating that multiphase CT images are feasible in predicting the recurrence of bladder cancer within 2 years after surgery. However, the efficacy of the combination model was still lower than 0.75, indicating that the radiomics model alone has limited application value in predicting the recurrence of bladder cancer within 2 years after surgery.

Therefore, this study also combined the clinicopathologic risk factors to construct a radiomics-clinical model to explore whether combining clinicopathologic risk factors could increase the efficacy in the prediction of the 2-year recurrence of bladder cancer. Some recent studies have shown (29, 30) that age, gender, grade, staging, surgical method, tumor size, and number of tumors are closely related to the recurrence of bladder cancer. The results of this study by using Cox univariate and multivariate proportional hazard models showed that the number, size, grade, staging of the tumor, and Rad-score were significantly correlated with the recurrence of bladder cancer, and the number, grade of the tumor, and Rad-score were the independent risk factors for the recurrence of bladder cancer. The increase in tumor number will lead to an increase in tumor load, and when the tumor number is large, it is difficult to completely remove the tumor by surgery, resulting in an increase in the recurrence rate. The different grades of tumors reflect the growth potential of tumors. High-grade tumors are prone to local invasion, increasing metastasis and recurrence risk. The radiomics-clinical model was constructed with the number of tumors, tumor grade, and Rad-score, of which the validation group AUC was 0.838, and its efficacy was better than that of the radiomics model. The calibration curve further demonstrated the effectiveness of its performance prediction. The decision curve analysis clearly showed that the radiomics-clinical model could obtain more clinical benefits than the radiomics model and could assist clinicians in personalized treatment and follow-up for patients with bladder cancer after surgery.

However, our study has the following limitations. First, since this study is retrospective, there may be inherent bias in the limited patient cohort from a single institution. Second, the sample size is relatively small, and the data from multiple centers are required to externally validate the overall performance of the model. Third, small follow-up duration is another limitation, since bladder cancer could be considered as a chronic disease; the follow-up should be extended. More importantly, due to the incomplete clinical database, this study did not include other potentially useful predictive factors, such as HER2 (31), Ki67 (29), and other immunohistochemical indexes. Moreover, preexisting clinical comorbidity like diabetes (32) can worsen outcomes in patients with high-grade T1 bladder cancer; it is a very important risk factor for the recurrence of bladder cancer. Furthermore, postoperative bladder infusion therapy also plays an important role in the prognosis of patients with bladder cancer, but different drugs have different effects (33–35). These predictors can be included in subsequent studies to explore whether they can increase the efficacy of bladder cancer recurrence prediction. Last but not least, although TURBT can eradicate an NMIBC completely, it commonly recurs and can progress to MIBC. Therefore, I think it is far from enough to focus only on recurrence. Whether the stage of recurrent tumor elevates, the degree of malignancy ascends, or distant metastasis occurs is crucial for the further treatment and prognosis of the patient. It is more important to diagnose a progression than a recurrence. Thus, this study still needs further improved and optimized before its implementation can be considered in clinical practice.

In conclusion, our results preliminarily demonstrate that radiomics features extracted from multiphase CT images, combined with important clinicopathologic risk factors, can predict the 2-year postoperative recurrence of bladder cancer. It still deserves optimization and further in-depth analysis with longer follow-up and wider sample size, which may be helpful for the early identification of patients with high recurrence risk, and for assistance and guidance of the formulation of individualized diagnosis and treatment plans in the future.

## Data Availability Statement

The original contributions presented in the study are included in the article/supplementary material. Further inquiries can be directed to the corresponding author.

## Author Contributions

JQ and SG collected and analyzed the data. JQ drafted the manuscript. ZL reviewed the pathological findings and provided the clinical data and followed up the patients. LY and SH revised the manuscript for important intellectual content. JY, HD and HS designed and supervised the study. All authors contributed to the article and approved the submitted version.

## Funding

This work was supported by the Jiangsu Medical Association Roentgen Imaging Research Special Fund Project: SYH-3201150-0017(2021012).

## Conflict of Interest

The authors declare that the research was conducted in the absence of any commercial or financial relationships that could be construed as a potential conflict of interest.

## Publisher’s Note

All claims expressed in this article are solely those of the authors and do not necessarily represent those of their affiliated organizations, or those of the publisher, the editors and the reviewers. Any product that may be evaluated in this article, or claim that may be made by its manufacturer, is not guaranteed or endorsed by the publisher.
